# The determinants of maternal perception of antenatal care services during the COVID-19 pandemic critical phase: A systematic review

**DOI:** 10.1371/journal.pone.0297563

**Published:** 2024-02-23

**Authors:** Nor Izyani Bahari, Rosnah Sutan, Zaleha Abdullah Mahdy

**Affiliations:** 1 Department of Public Health Medicine, Faculty of Medicine, Universiti Kebangsaan Malaysia, Bandar Tun Razak, Kuala Lumpur, Malaysia; 2 Department of Obstetrics and Gynaecology, Faculty of Medicine, Universiti Kebangsaan Malaysia, Bandar Tun Razak, Kuala Lumpur, Malaysia; Kasturba Medical College Mangalore, Manipal Academy of Higher Education, INDIA

## Abstract

**Introduction:**

The COVID-19 pandemic has exerted devastating effects on healthcare delivery systems, specifically those for pregnant women. The aim of this review was to determine the maternal perception of antenatal health care services during the COVID-19 pandemic critical phase.

**Methods:**

Scopus, Web of Science, SAGE, and Ovid were systematically searched using the keywords “maternal”, “COVID-19 pandemic”, “maternal health service”, and “maternal perception”. Articles were eligible for inclusion if they were original articles, written in English, and published between January 1, 2020, and December 12, 2022. This review was performed based on the Preferred Reporting Items for Systematic Reviews and Meta-Analyses guidelines. Eligible articles were assessed using the Mixed Methods Appraisal Tool. Thematic analysis was used for data synthesis.

**Results:**

Of 2683 articles identified, 13 fulfilled the inclusion criteria and were included in the narrative synthesis. Five themes emerged regarding the determinants of maternal perception of antenatal healthcare services during the COVID-19 pandemic critical phase: lack of psychosocial support, poor maternal healthcare quality, poor opinion of virtual consultation, health structure adaptation failure to meet women’s needs, and satisfaction with maternal health services.

**Conclusion:**

Maternal perception, specifically pregnant women’s psychosocial and maternal health needs, should be focused on the continuation of maternal care during the COVID-19 pandemic. It is critical to identify the maternal perception of maternal health services during the pandemic to ensure health service equity in the “new normal” future.

## Introduction

The novel coronavirus outbreak (COVID-19) began at the end of 2019 when multiple pneumonia cases of unknown etiology were reported in Wuhan, China. The World Health Organization (WHO) subsequently declared COVID-19 to be a Public Health Emergency of International Concern and subsequently, a global pandemic was declared in March 2020 [1]. The critical phase of the COVID-19 pandemic refers to the period during which many countries applied wide-ranging movement controls or restrictions (“lockdowns”) and measures for physical distancing [2]. These stringent measures undoubtedly exerted a substantial adverse effect on individual persons, neighborhoods, and societies. Nevertheless, many governments deemed such measures crucial to limit the transmission of COVID-19 [2]. The maternal perception of Antenatal Care (ANC) services during the critical phase would clearly differ from before the pandemic phase was announced and clearly exerted more adverse effects. To date, as of June 7th, 2023, there were 767,750,853 COVID-19 cases were confirmed globally, which included 6,941,095 deaths [3].

Overall ANC services are unaltered during the COVID-19 pandemic [4]. Nevertheless, providers of maternal care should be cognizant of the greater risks for domestic violence and mental health problems, given the social and economic impact of the pandemic. With the occurrence of COVID-19 in the community, increased stress and tension are likely to occur among pregnant women and persons surrounding themselves [4]. Such concerns can exacerbate pregnancy stresses and risks. The focus on COVID-19 a new alert may divert the opinions of pregnant women regarding the importance of routine ANC. Accordingly, maternal health services should take precedence as essential core health services. The monitoring of ANC during the pandemic should be continued as usual especially those pregnant women with identified risk factors to ensure safe motherhood and delivery.

Most populations have been critically affected by the pandemic, especially those in countries with under-resourced health systems [5]. The effects of COVID-19 on livelihoods and the increased morbidity and mortality due to COVID-19 have imposed an increased burden on the health system [5]. Deployment of health staff during the pandemic (due to quarantining following COVID-19 exposure or assignation to screening and vaccination centers) has affected routine maternal healthcare services. Clinic visits arrangements to minimize COVID-19 transmission risk among pregnant women and medical staff during antenatal visits resulted in a reduction in antenatal appointments [6, 7].

Therefore, vulnerable populations such as high-risk expectant women were required to attend modified maternal health care services [8] and follow the relevant guidelines to continue receiving ANC services at selected facilities. Based on these additional challenges, it is essential to determine the factors that influence pregnant women’s uptake of modified maternal services during the COVID-19 pandemic. The aim of the present review was to analyze the existing literature on the determinants of pregnant women’s perception of maternal healthcare services during the critical phase of the COVID-19 pandemic.

## Materials and methods

The Preferred Reporting Items for Systematic Reviews and Meta-Analyses (PRISMA) guidelines were used in this review [9]. PRISMA Checklist is available as supporting information (S1 Table). The study protocol is not registered with PROSPERO for systematic review registration.

### Research question

The research question for the review was structured based on elements of the Population, Phenomena of interest, Context, and Outcome (PICo approach) [10]. Based on PICo, we defined the population of interest as the antenatal women; a phenomenon of interest is the maternal health services; the context is the pandemic COVID-19 critical phase; and the outcome of interest is the maternal perception of ANC services. The main research question was “What is the maternal perception of ANC services during the COVID-19 critical phase?”

### Systematic search

**Fig 1:**
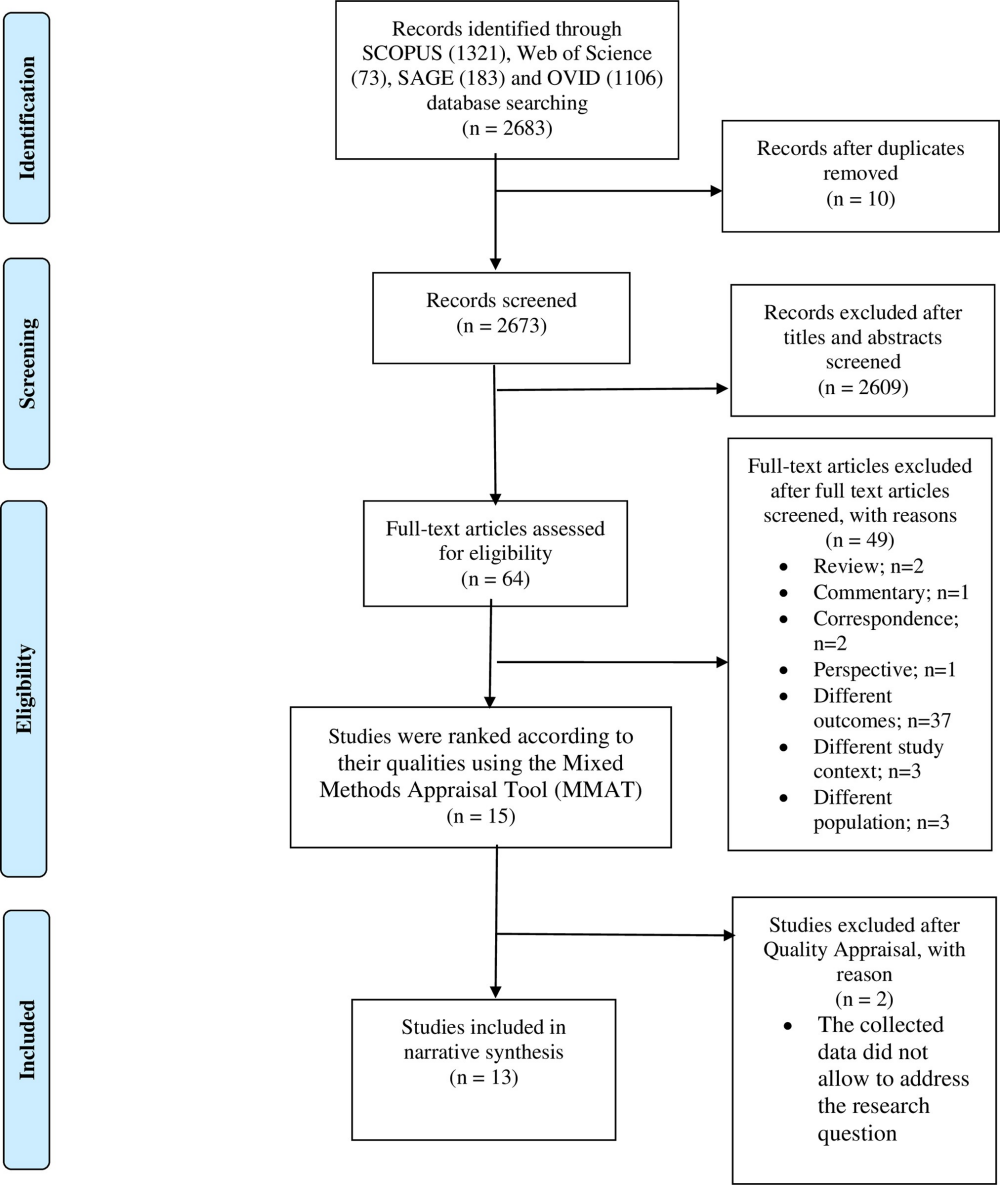
PRISMA flow diagram.

The three main processes in the systematic search strategy were identification, screening and eligibility evaluation. Fig 1 depicts the PRISMA flow diagram of the study.

The literature search was performed on 12th December 2022, and involved articles published between January 1, 2020, and December 12, 2022, from the Scopus, Web of Science, SAGE, and Ovid databases. Articles were searched using synonyms, related terms, and variations of the main keywords. The search terms utilized across all databases for the study were: (“mother” OR “maternal” OR “antenatal mother” OR “pregnant mother” OR “expecting mother” OR “expectant” OR “gestating” OR “gravid” OR “parous”) AND (“COVID-19” OR “COVID” OR “coronavirus” OR “2019 novel coronavirus” OR “SARS-CoV-2” OR “pandemic” OR “COVID-19 virus infection” OR “2019-nCoV infection” OR “coronavirus disease 2019” OR “SARS CoV 2 infection” OR “COVID-19 pandemic” OR “Severe Acute Respiratory Syndrome Coronavirus 2” OR “Wuhan Coronavirus” OR “SARS Coronavirus 2”) AND (“maternal health service” OR “health service” OR “maternal health”) AND (“perception” OR “knowledge” OR “opinion” OR “thought” OR “viewpoint” OR “awareness” OR “attitude” OR “discernment” OR “insight” OR “perceptiveness” OR “perceptivity” OR “understanding”). Supporting information as shown in S2 Table presents a detailed analysis of the search strategy. The search was enriched using Boolean operators and phrase searching. A total of 2683 articles were retrieved for screening.

Of the initial 2683 articles, 10 duplicate articles were removed, leaving 2673 articles for title screening. The inclusion criteria were: (1) open access journal article written in English and published between 1st January 2020 and 12th December 2022, (2) an original article (3) a quantitative, qualitative, mixed-method study, (4) the context of an article based on maternal health service focused on COVID-19 during the critical phase, (5) article outcome emphasized on maternal perception of ANC services during the COVID-19 critical phase, and (6) article focused on antenatal women population. The exclusion criteria were: (1) in vivo/in vitro study, (2) grey literature (report, commentary, letter to editor), and (3) animal study. If the title screening outcome was uncertain, the abstract was read to determine whether the article was to be included or excluded. A total of 2609 articles were removed during the title and abstract screening, as they did not fulfill the inclusion criteria, leaving 64 articles to undergo the full-text assessment and eligibility evaluation.

Two researchers (NIB, and RS) jointly evaluated the eligibility of the 64 articles. Full-text articles were retrieved and distributed accordingly among the two authors. The third author (ZAM) was consulted when any discrepancies in reviews appeared. The present review included articles that focus on (1) pregnant women’s perceptions, (2) ANC services during the COVID-19 critical phase, (3) original research, and (4) clear methodology. Subsequently, only 15 articles remained for quality appraisal and proceed to data synthesis.

### Quality appraisal

The Mixed Methods Appraisal Tool, version 2018 (MMAT) [11] was used to assess all 15 studies for critical appraisal. The MMAT is efficient and practical for evaluating the quality of various study designs including quantitative, qualitative, and mixed-method. The MMAT has undergone validation and is widely used for systematic reviews, where both quantitative and qualitative studies were included. The tool was considered applicable for this review given that it enables the assessment of empirical literature (quantitative, mixed-method, qualitative), efficient to use one tool for concomitantly appraising studies for quality assessment as it is valid and reliable [11]. The narrative analysis was conducted with a pre-designed data extraction form to acquire all pertinent data [11]. The criteria for evaluating the methodology in each study were determined using the structured approach framework described by Hong *et al*. [11]. Subsequently, the MMAT evaluations were based on five criteria sets, where each criterion was graded as pass (1) or fail (0). The five criteria sets were based on Hong et al. [11]: (a) Is the research question appropriately answered with the study approach? (b) Is the research question adequately addressed by the methods of collecting the outcome of interest? (c) Did the data yield adequate findings? (d) Did the data verify the interpretation of the results satisfactorily? (e) Are the sources, collection, analysis, and interpretation of the data coherent? For each criterion, we need to rate it as ‘yes’, ‘no’, or ‘can’t tell’ based on how the appropriateness of information is available in the articles.

The authors of the MMAT tool did not specify the cut-off scores that distinguish high- and low-quality studies. Nevertheless, reviewers performing quality evaluations are advised to determine their own parameters [12]. As the MMAT was developed relatively recently, there is a dearth of MMAT research specifically targeting maternal health during the COVID-19 pandemic. Accordingly, we established that studies that scored ≥60% were considered satisfactory and incorporated into the analysis [12]. For example, the total score of a study that met four of five MMAT criteria would be 80% and would be included in this review. Two reviewers (NIB and RS) assessed the articles to reach an agreement on rating before including articles in the review. Any discrepancies between the two reviewers were discussed for consensus. Disagreements were examined with a third reviewer (ZAM) before the final decision was made. The evidence synthesis in the MMAT appraisal table is listed (S3 Table, Supporting Information). Two studies were excluded for reasons that were unclear in the methodology to respond to the research question addressed, therefore leaving 13 studies. Eleven studies met all MMAT criteria (100%), while two studies received a score of 80% and 60%, respectively (S3 Table, Supporting Information). A description of the quality of the studies was included to ensure the transparency of the review without focusing only on the quality percentages assessed alone.

### Data extraction and analysis

Thematic analysis was used in the present systematic review to synthesize and integrate the research findings according to consensus scopes derived from the literature [13]. All included articles were reviewed comprehensively before the findings were extracted to an Excel sheet. The extracted data patterns were studied and categorized into themes that were discussed to determine if the themes were appropriate for the present systematic review. The themes of interest were:

- Psychosocial support
- Maternal healthcare/service quality
- Telehealth/virtual consultation
- Healthcare adaptation during a pandemic
- Satisfaction with maternal health service

## Results

### Background of the selected articles

A total of 13 studies were included in this systematic review (Table 1). There was only one study published in 2020 [14], seven studies in year 2021 [8, 15–20] and five studies were published in the year 2022 [21–25]. Two studies were conducted in Ethiopia [8, 18], the United Kingdom [14, 23], and Australia [17, 20], and only one study from the United States [15], Turkey [24], Iran [22], India [21], and Malaysia [19]. The remaining two studies involved multiple European countries [16], and British Columbia [25]. Five studies were quantitative (cross-sectional) [8, 16, 18, 19, 24], five were qualitative study design using thematic analysis [14, 15, 21, 22, 25], and three studies were mixed-method design (one convergent parallel design and two explanatory sequential design) [17, 20, 23]. Two studies involved only pregnant women among their study sample recruitment [22, 24], six studies involved both pregnant and postpartum women [14, 16, 18, 20, 23, 25], and five studies involved pregnant women and their partners, healthcare workers including the midwives, medical practitioners, and midwifery students who provided maternity care [8, 15, 17, 21, 23].

**Table 1:** Characteristics of included studies.

No.	Title, author, publication year, country	Quality evaluation	Participants	Methodology	Outcome
1.	Women’s perceptions of COVID-19 and their healthcare experiences: a qualitative thematic analysis of a national survey of pregnant women in the United Kingdom, Karavadra *et al*. (2020), United Kingdom	100%	1451 (pregnant women, n = 1221; postpartum women, n = 230)	Qualitative (open-ended data from a survey underwent thematic analysis)	Mothers considered virtual telehealth as “impersonal care” and stated that it influenced the security of information provided to their healthcare workers. They considered reviewing sensitive issues or mental health concerns over the telephone awkward and inappropriate.
2	Where the system failed: the COVID-19 pandemic’s impact on pregnancy and birth care Altman *et al*., (2021), United States	100%	29 (pregnant women, n = 15; healthcare providers, n = 14)	Qualitative (thematic analysis)	Virtual telehealth was expensive. The human connection was absent from antenatal and postpartum online visits. The mothers perceived that they received poor encouragement from the care providers.
3.	Vaccine willingness and impact of the COVID-19 pandemic on women’s perinatal experiences and practices: A multinational, cross-sectional study covering the first wave of the pandemic Ceulemans *et al*. (2021), Ireland, Norway, Switzerland, the Netherlands, United Kingdom, Belgium	100%	16 063 (pregnant women, n = 6661; postpartum women, n = 9402)	Quantitative (cross-sectional)	The pregnant women were dissatisfied due to partners being absent from antenatal check-ups and ultrasounds. They reported that midwives, general practitioners, and obstetricians conducted antenatal follow-up less frequently.
4.	Exploring COVID-19 related factors influencing antenatal care services uptake: a qualitative study among women in a rural community in Southwest Ethiopia Hailemariam *et*.*al*. (2021), Ethiopia	100%	53 (pregnant women, n = 44; healthcare providers, n = 9)	Quantitative (cross-sectional)	Pregnant women were dissatisfied by the lack of support material and staff during ANC visits. They perceived that the health facilities were considered potential COVID-19 infection sources.
5	Experiences of receiving and providing maternity care during the COVID-19 pandemic in Australia: A five-cohort cross-sectional comparison Bradfield *et al*. (2021), Australia	60% (confounders not controlled, response rate not mentioned)	3701 (pregnant and postpartum women, n = 2840; partners, n = 92; midwives, n = 560; medical practitioners, n = 78; midwifery students, n = 31)	Mixed-method (convergent parallel)	Pregnant women feared of contracting COVID-19 when receiving service. Infection might have been influenced by healthcare provider transmission and lack of clean water or sanitizer at the health facility.
6.	Maternal health care services utilization amidst COVID-19 pandemic in West Shoa zone, central Ethiopia Temesgen *et al*. (2021), Ethiopia	100%	844 (pregnant women, n = 329; postpartum women, n = 657)	Quantitative (cross-sectional)	The women feared of catching COVID-19 when receiving service. Healthcare provider transmission and lack of clean water or sanitizer at the health facility might have influenced infection.
7	Pregnancy and COVID-19 Pandemic Perception in Malaysia: A Cross-Sectional Study Syed Anwar Aly *et al*. (2021), Malaysia	100%	415 (pregnant women, n = 240; postnatal women, n = 175)	Quantitative (cross-sectional)	The majority of pregnant women stated that the pandemic did not affect their antenatal appointments. Government clinics or private general practitioners were perceived as helpful and very supportive.
8.	Can positive mindsets be protective against stress and isolation experienced during the COVID-19 pandemic? A mixed methods approach to understanding emotional health and wellbeing needs of perinatal women Davis *et al*. (2021), Western Australia	100%	174 (pregnant women, n = 31; postpartum women, n = 143)	Mixed-method (explanatory sequential)	The women were most concerned was the absence of service access. A long duration was required for physical health services (ultrasound scans) or telephone or telehealth consultations replaced them. Anxiety increased by cancellations and delays the services appointment. The absence of clear communication regarding pregnancy greatly upset the majority of women. Authors recommended that the information should be conveyed in a clearly comprehensible manner.
9	Accessing Antenatal Care (ANC) services during the COVID-19 first wave: insights into decision-making in rural India Bankar and Ghosh (2022), India	100%	29 (pregnant women, n = 12; healthcare provider, n = 17)	Qualitative (thematic analysis)	Pregnant women were concerned about catching up COVID-19 from physical visits while receiving ANC services at the health center. Public health systems were not trusted. Healthcare workers provided inadequate service delivery frequency. The usefulness of interactions was limited due to the lack of in-person visits with health practitioners.
10.	High-risk pregnant women’s experiences of the receiving prenatal care in COVID-19 pandemic: a qualitative study Mirzakhani *et al*. (2022), Iran	100%	31 pregnant women	Qualitative (thematic analysis)	Health services were refused by pregnant women who were afraid of catching up COVID-19. COVID-19 infection could lead to social pressures (spousal and familial intimidation) if infected with COVID-19. Staff behaviors were considered unsupportive when discussing COVID-19 infection in front of the pregnant women (no transparency of information). Women with high-risk pregnancies felt increased anxiety, stress, and fear when COVID-19 deaths were reported in front of them, prompting their decision to depart from the hospital.
11.	Expectant parents’ perceptions of healthcare and support during COVID-19 in the UK: a thematic analysis Aydin *et al*. (2022), United Kingdom	80% (sample number was not representative of the target population)	507 (pregnant women, n = 1; postpartum women, n = 503; partners, n = 3)	Mixed-method (explanatory sequential)	Pregnant women considered health care services unsupportive when appointments were fewer and rushed, highlighting the poor communication and confusion. Observed insensitivity (inconsiderate staff treatment of patients) denoted failure to fully resolve mental health concerns. The absence of online and personal interactions and partner’s absence were the main contributors to maternal anxiety.
12.	Expectations of pregnant women for antenatal care services and factors affecting anxiety severity during the COVID-19 pandemic Kumru *et al*. (2022), Turkey	100%	447 pregnant women	Quantitative (cross-sectional)	Pregnant women most frequently worried about catching up COVID-19 from other patients during hospital follow up or delivery. The women felt that perinatal COVID-19 infection would harm their babies, followed by concern that their relative or spouse would be absent during the delivery. Up to 50% wanted to escape the follow-up as they were concerned about catching up COVID-19 from hospital.
13.	Rural Residents’ Perinatal Experiences During the Initial Months of the COVID-19 Pandemic: A Qualitative Study in British Columbia Sullivan *et al*. (2022), British Columbia	100%	16 (pregnant women, n = 7; postpartum women, n = 9)	Qualitative (thematic analysis)	The participants’ biggest trial was the absence of social support outside and in the hospital or care center. Altered guidelines that resulted in partners being prevented from accompanying mothers during perinatal follow-ups exacerbated mothers’ opinions of healthcare service. Frustration concerning the substantial reduction of in-person care frequency changes (ultrasound appointments) to curtail unnecessary clinic visits. Mother–care provider relationship advantages grown over time emphasized perinatal care providers “going above and beyond” to alleviate the stressful effects of the pandemic.

### Themes

Five themes emerged from the 13 included studies on the maternal perceptions of ANC services during the COVID-19 pandemic critical phase (Table 2): psychosocial support, maternal healthcare quality, telehealth or virtual consultation, healthcare adaptation during the pandemic, and satisfaction with maternal health service.

**Table 2:** Emerged themes on maternal perception of ANC service during the COVID-19 pandemic critical phase.

Maternal Perception (Theme)	Articles												
	1	2	3	4	5	6	7	8	9	10	11	12	13
	Article Reference ID												
	Karavadra *et al*. (2020)	Altman *et al*. (2021)	Ceulemans *et al*. (2021)	Hailemariam *et al*. (2021)	Bradfield *et al*. (2021)	Temesgen *et al*. (2021)	Syed Anwar Aly *et al*. (2021)	Davis et al. (2021)	Bankar & Ghosh (2022)	Mirzakhani et al.(2022)	Aydin et al. (2022)	Kumru et al. (2022)	Sullivan et al (2022)
1. Psychosocial support			*	*					*	*	*	*	*
2. Quality in maternal healthcare/service				*	*	*		*			*		*
3. Telehealth/virtual consultation	*							*					
4. Fulfill maternal demand		*	*					*	*				
5. Maternal positive satisfaction							*						*

* mentioned in the article

#### Theme 1: Psychosocial support

Seven studies [8, 16, 21–25] determined that a lack of psychosocial support influenced maternal perceptions of ANC service during the COVID-19 critical phase. The main factor that negatively affected the experience was the absence of the women’s partners during ANC visits, as chaperones were not permitted at routine checkups during the pandemic critical phase. Moreover, pregnant women’s anxiety or stress might have been aggravated by the requirement to maintain social distancing and caution when interacting with others during health clinic visits and contact with healthcare workers, who were COVID-19 front-liners [16].

Most pregnant women were also anxious about practicing social isolation following their visit to the health facility as they might be viewed as virus spreaders in the community. Subsequently, they feared that others might avoid meeting with them for fear of contracting COVID-19, which created a social stigma. Some women felt anxious about caring for their children following visits to healthcare facilities as they were uncertain about whether they had been infected with COVID-19. A few women declined to know about their COVID-19 screening results as they could not tolerate the stress of knowing if they had contracted COVID-19 [8].

#### Theme 2: Maternal healthcare quality

Six studies [8, 17, 18, 20, 23, 25] highlighted the perceived poor quality of maternal healthcare services. Bradfield *et al*. [17] reported that pregnant women were generally dissatisfied with the health service modifications during the COVID-19 pandemic critical phase. However, a small proportion of the women received services in a timely manner and the care needed during the critical phase [17]. Some pregnant women perceived poorer quality of care during the critical phase due to staff and material shortages. Moreover, many pregnant women reported maltreatment by disrespectful healthcare providers. Lastly, pregnant women perceived health facilities as a COVID-19 infection risk due to the overcrowded environment that predisposed them to COVID-19 infection while receiving ANC care. Nevertheless, most women felt that the benefit of ANC services to themselves and their babies outweighed the risk of contracting COVID-19 [8]. In the study conducted in Ethiopia, pregnant women were reluctant to utilize health services due to the fear of contracting COVID-19 while receiving health services. They also feared contracting COVID-19 from healthcare providers, particularly in poor-hygiene health facilities that lacked sanitization [18].

#### Theme 3: Telehealth or virtual consultation

Poor opinion of virtual consultation influenced pregnant women’s perception of maternal health services during the critical phase of the pandemic [14, 20]. Most women perceived that virtual consultations provided impersonal care and were reluctant to disclose too much information to their healthcare workers. Women with a history of high-risk pregnancy preferred face-to-face consultation to reduce their anxiety. Other women were embarrassed about discussing their mental and social health problems online as they perceived that discussing personal issues over the telephone was inappropriate [14].

#### Theme 4: Healthcare adaptation during pandemic

The failure of healthcare adaptation during the COVID-19 pandemic critical phase to meet pregnant women’s needs was reported in four studies [15, 16, 20, 21]. Telehealth visits were implemented during the critical phase to reassure pregnant women and connect them to ANC services. However, many women perceived that this type of approach was not well fulfilled their needs during the critical phase [15]. The high levels of perceived lack of human connection with healthcare workers during virtual health visits were influential in the management aspect of ANC and postpartum care. Furthermore, some women felt that telehealth ANC visits were impractical and costly.

Most pregnant women also identified a change in care whereby they perceived that healthcare providers managed them as a contact of COVID-19 exposure. Some women perceived a feeling of inadequate care during their hospital stays, which stemmed from the lack of healthcare providers checking to determine if they needed medication or further management [15, 16]. The American study highlighted the relationship between the COVID-19 effect on maternal health care because of racism and discrimination, which was magnified during the pandemic critical phase when increased incidences of disrespectful care towards antenatal women of color were observed [15].

#### Theme 5: Satisfaction with maternal health service

Positive perception by pregnant women as satisfaction with the maternal health services received during the critical phase was mentioned in two studies [19, 25]. In the Malaysian study, it was reported that government or private clinic doctors or nurses were very supportive and helpful [19]. The pregnant women’s appointments were not affected by the critical phase and they continued to receive ANC as scheduled.

## Discussion

The perceptions of pregnant women regarding maternal health services during the COVID-19 pandemic critical phase was reviewed. In this review, the inclusion criteria and research scope focus on the observed organizational and psychosocial outcomes. The COVID-19 outcome was mainly linked to the pregnant women’s perception of psychological well-being, the effectiveness of telehealth consultation, and the quality of maternal health service.

The frequent emerging theme was the absence of psychosocial encouragement [15, 16, 26]. This theme was found paralleled with a study done in Ireland, which reported that pregnant women received poor psychosocial assistance after the presence of partners was restricted during regular appointments and delivery [26]. Pregnant women experienced psychological distress (anxiety or fear) stemming from social isolation when their partners or husbands were prevented from accompanying them to ANC visits [26]. Women with first pregnancies (primigravidae) reported more intense psychological distress given that they had never received maternal healthcare services previously and were unaccustomed to the routine [26]. Nonetheless, stringent policies were required to avert congestion in enclosed spaces such as maternal health clinics to decrease the risk of COVID-19 spread [26].

A study performed in Turkey reported that pregnant women experienced intensified symptoms of anxiety during the pandemic critical phase [27]. As the women were uncertain and unclear regarding the risk to mother and child health during this phase, they weighed the situation lightly and also received inadequate information. The women reported feelings of fear and anxiety as COVID-19 cases mounted given the wide community spread [27]. Another study conducted in Israel mentioned that psychological distress among pregnant women was positively associated with the fear of being infected with COVID-19 [28]. Moreover, maternal healthcare follow-up might have been influenced by the apparent absence of social support and an unsatisfactory relationship between the patient and the doctor [29]. Women who experience perinatal loss demonstrate emotional suffering, which is assuaged by support from friends. Therefore, constant postnatal emotional support from immediate family members, spouse or partner, and close friends is necessary when women receive unsatisfactory ANC.

This review revealed that pregnant women’s opinions on access to healthcare services during the critical phase of COVID-19 were influenced by poor-quality maternal health services [8, 17, 18, 20, 23, 25]. The movement restriction approach for restricting the spread of COVID-19 greatly affected the delivery of health services. While there were varying opinions regarding the disruption level, the effect was most evident in medicine supply, maternity services, and immunization [30]. This result was in accordance with the Irish study, in which the pregnant women’s opinions of poor-quality essential service during antenatal visits were influenced by the lack of information and poor communication by medical staff [26]. Furthermore, it was precipitated by feelings of suspicion and doubt regarding maternal care resulting from the cancellation or postponement of numerous appointments, including the ultrasound scan [26].

In this review, the negative opinion of pregnant women about consultations of virtual health during the COVID-19 pandemic critical phase was emphasized [14, 20]. Other studies reported overall satisfaction regarding virtual prenatal care during the pandemic. The pregnant women in those studies tended to be satisfied with their pandemic virtual prenatal care experiences. Nevertheless, they also generally favored in-person care in the absence of a pandemic. In-person visits might be preferred, as they establish more optimal conditions to become familiar with the health care provider and present the opportunity for a more thorough physical examination (routine measurement of blood pressure, fetal heartbeat detection), which virtual prenatal care might limit [31].

These poor opinions contradicted the results of an Australian study, where ANC that integrated telehealth decreased in-person consultation by 50% without adversely affecting the outcomes of pregnancy [32]. There were no significant differences linked to adverse outcomes of pregnancy, such as restriction of fetal growth, pre-eclampsia-complicated pregnancy, gestational diabetes mellitus, and stillbirth, when telehealth was used [32]. This result is expected able to provide evidence to persuade pregnant women to undergo ANC follow-up via online platforms during the COVID-19 pandemic to ensure continuity of maternal care and reduce physical visits to the clinic. Nevertheless, prenatal care clinics should not reject the concept of virtual components as a standard of care, where a hybrid or virtual model design should be considered carefully via constant examination and development to guarantee its success [31]. According to the articles included in this review, the digital health system method should develop beyond emergency obstetric care to incorporate early and preventive interventions with good assimilation [33]. Using digital platforms to provide complete and cohesive care antenatally and postpartum is vital to generate preparedness for social change and support maternal health-seeking behavior [33].

In this review, the healthcare adaptation failure during the COVID-19 pandemic critical phase was found inability to fulfill maternal demand for ANC services [15, 16, 20, 21]. The global sentiment of being overwhelmed subsequent to the COVID-19 effect on maternal health services was especially obvious in the form of staff unavailability, limited anti-tetanus toxoid (ATT) vaccine supply for pregnant women, routine antenatal clinics closures, and vaccination clinics suspensions. Comparable doubts were expressed by pregnant women from low-, middle-, and high-income countries after the COVID-19 pandemic critical phase [34]. A qualitative study conducted in Iran reported that pregnant women stated that they felt vulnerable and unsupported when healthcare services were disrupted early in the epidemic; where pregnancy-related arrangements were canceled, specialist private offices closed down, and hospital visit wait times were drastically extended. Subsequently, the women experienced extreme stress during the pandemic outbreak [35]. Therefore, it is crucial that maternal healthcare service policies be enhanced during the pandemic to assure pregnant women’s challenges and to fulfill their demands.

The Malaysian study reported that pregnant women expressed high satisfaction with the maternal healthcare services received during the pandemic critical phase [19]. Nevertheless, the study was performed during the initial pandemic stages, when Malaysian maternal healthcare services were not severely affected. There was no ANC appointment rescheduling during the initial phase of the pandemic. Furthermore, the major difference was that the health clinics required pregnant women to adhere to COVID-19 standard operating processes. However, the Malaysian situation deteriorated since then, where there were markedly increased cases of COVID-19, and intensive care unit admission was required by many pregnant women. Thus, pregnant women’s opinions of maternal healthcare services might vary according to the most recent COVID-19 condition.

Likewise, a study performed in Pakistan reported that approval of pre-delivery care services was related to lower fear of catching COVID-19 [36]. The findings indicated that women were more conscious of COVID-19 safety protocols at registration and admission. Hospitals with more obvious COVID-19 prevention procedures must aid in reducing pregnant women’s fear at the beginning and render their hospital stay less anxious. The observable signs for preventive safety include sterilization at entrances, masking being compulsory, physically distanced seating areas, frequent institutional sanitation and hand sanitizer availability, and personal protective equipment (PPE) kits for healthcare staff (36).

### Recommendations

As maternal health service quality has been compromised due to health staff mobilization to assist in COVID-19 management, health organizations must implement contingency plans to ensure that essential services are not disrupted. For example, maternal health services must be prioritized to ensure that pregnant women can access proper antenatal treatment to safeguard maternal and fetal health. The comparisons of face-to-face and virtual ANC consultations yielded insufficient evidence on pregnancy outcomes. Nevertheless, health authorities should continue to promote virtual telehealth uptake among pregnant women, especially in underprivileged areas, to evaluate its short- and long-term effectiveness. Furthermore, policies that limit partners during antenatal follow-up and birth can potentially exacerbate mental health issues and should be implemented with sensitivity. Therefore, health organizations should consider a mandatory policy that integrates mental health services in any outbreak or disaster preparedness program.

### Limitations

Most of the studies included were cross-sectional studies. Therefore, a causal-effect relationship could not be determined. A qualitative study is recommended for future research so that pregnant women’s perceptions of their health needs during the COVID-19 pandemic can be explored in greater depth. This review focused on published open-access literature. Thus, unpublished research and paywalled research could have been overlooked.

## Conclusion

The COVID-19 crisis has significantly affected health services for pregnant women. There was an increased risk of psychological distress during the critical phase of the pandemic. Strengthening a coordinated approach during outbreak management is crucial and timely. As digital telehealth consultation minimizes the COVID-19 transmission risk, its application should be promoted. The determinants of pregnant women’s perception of maternal healthcare services during the COVID-19 pandemic critical phase can guide policymakers’ evaluation of pregnant women’s need to access maternal healthcare services during the pandemic.

## Supporting information

S1 Table PRISMA checklist. (PDF)

S2 Table Mesh words used in the search process. (PDF)

S3 Table Quality appraisal using the Mixed Methods Appraisal Tool (MMAT). (PDF)
