# Supplementary material for: The determinants of maternal perception of antenatal care services during the COVID-19 pandemic critical phase: A systematic review
Source: PLoS One. 2024 Feb 23;19(2):e0297563. doi: 10.1371/journal.pone.0297563 (PMC10889657; doi:10.1371/journal.pone.0297563)
Supplement: S2 Table — (PDF) [file pone.0297563.s002.pdf]

Table 1. Mesh words used in the search process

Database	Search string
SCOPUS	TITLE-ABS-KEY ("mother*" OR "maternal" OR "antenatal mother*" OR "pregnant mother*" OR "expecting mother*" OR "expectant*" OR "gestating" OR "gravid" OR "parous") AND ("COVID-19" OR covid OR coronavirus OR "2019 novel coronavirus" OR "SARS-CoV-2" OR pandemic OR "COVID-19 virus infection" OR "2019-nCoV infection" OR "coronavirus disease 2019" OR "SARS CoV 2 infection" OR "COVID-19 pandemic" OR "Severe Acute Respiratory Syndrome Coronavirus 2" OR "Wuhan Coronavirus" OR "SARS Coronavirus 2") AND ("maternal health service*" OR "health service*" OR "maternal health*") AND (perception* OR knowledge* OR opinion* OR thought* OR "view point*" OR awareness OR attitude* OR discernment OR insight OR perceptiveness OR perceptivity OR understanding)
Web of Science	TS=((mother OR maternal OR "antenatal mother" OR "pregnant mother" OR "expecting mother" OR "expectant" OR "gestating" OR "gravid" OR "parous") AND ("COVID-19" OR COVID OR coronavirus OR "2019 novel coronavirus" OR "SARS-CoV-2" OR pandemic OR "COVID-19 virus infection" OR "2019-nCoV infection" OR "coronavirus disease 2019" OR "SARS CoV 2 infection" OR "COVID-19 pandemic" OR "Severe Acute

	Respiratory Syndrome Coronavirus 2" OR "Wuhan Coronavirus" OR "SARS Coronavirus 2") AND ("maternal health service" OR "health service" OR "maternal health") AND (perception OR knowledge OR opinion OR thought OR "viewpoint" OR awareness OR attitude OR discernment OR insight OR perceptiveness OR perceptivity OR understanding))
OVID	("mother*" or "maternal" or "antenatal mother*" or "pregnant mother*" or "expecting mother*" or "expectant*" or "gestating" or "gravid" or "parous") and ("COVID-19" or COVID or coronavirus or "2019 novel coronavirus" or "SARS-CoV-2" or pandemic or "COVID-19 virus infection" or "2019-nCoV infection" or "coronavirus disease 2019" or "SARS CoV 2 infection" or "COVID-19 pandemic" or "Severe Acute Respiratory Syndrome Coronavirus 2" or "Wuhan Coronavirus" or "SARS Coronavirus 2") and ("maternal health service*" or "health service*" or "maternal health*") and (perception* or knowledge* or opinion* or thought* or "viewpoint*" or awareness or attitude* or discernment or insight or perceptiveness or perceptivity or understanding)
SAGE	("mother*" OR "maternal" OR "antenatal mother*" OR "pregnant mother*" OR "expecting mother*" OR "expectant*" OR "gestating" OR "gravid" OR "parous") AND ("COVID-19" OR COVID OR coronavirus OR "2019 novel coronavirus" OR "SARS-CoV-2" OR pandemic OR "COVID-19 virus infection"

	OR "2019-nCoV infection" OR "coronavirus disease 2019" OR "SARS CoV 2 infection" OR "COVID-19 pandemic" OR "Severe Acute Respiratory Syndrome Coronavirus 2" OR "Wuhan Coronavirus" OR "SARS Coronavirus 2") AND ("maternal health service*" OR "health service*" OR "maternal health*") AND (perception* OR knowledge* OR opinion* OR thought* OR "viewpoint*" OR awareness OR attitude* OR discernment OR insight OR perceptiveness OR perceptivity OR understanding)

Table 2. Table of evidence

No	Author, Country, Year of Publication	Quality assessment	Participants	Method	Intervention/Outcome
1	Altman *et al.* [11] United States, 2021	100%	29	Qualitative	Adaptations to the healthcare structures during COVID 19 failed to meet maternal healthcare needs. Mother has perceived that virtual telehealth was costly and virtual visits during pregnancy and postpartum period were lacked human connection, resulting in poor reassurance from their providers. Emphasis on the need to address racism and discrimination leads to disrespectful care. The discrimination felt magnified during the pandemic.

2	Ceulemans *et.al.*[15] Ireland, Norway, Switzerland, the Netherlands, and United Kingdom, Belgium 2021	100%	16,063	Quantitative	Dissatisfaction arose from the partner's absence during antenatal check-ups and ultrasounds and less frequent antenatal follow-up by the midwife, general practitioners, and obstetricians. The antenatal mothers reported anxiety or stress due to social isolation and poor health information support received during the pandemic.
3	Hailemariam *et.al.*[4] Ethiopia 2021	100%	44	Qualitative	The majority perceived the quality of maternal health services during the COVID19 pandemic as poor. Shortage of staff and support material during COVID-19 led to dissatisfaction with ANC

					visits. Some claimed to have encountered maltreatment and disrespect from the healthcare providers. Mothers felt anxiety about social isolation after they visited health facilities because they might be considered to bring the virus into the community, and others would refrain from meeting them. Various psychological and social support and costs of being quarantined impacted the care of their children. It led to a reluctance to know their screening result due to the inability to withstand the stress of positive coronavirus tests. Mothers perceived that

					the health facilities were the potential source of COVID 19 infection. Thus, exposing them to unhygienic services.
4	Karavadra *et al.*[12] United Kingdom 2020	100%	1451	Qualitative	Mothers perceived virtual telehealth as 'impersonal care' and claimed it affected the security of information disclosed to their healthcare workers. They felt embarrassed and inappropriate to discuss mental health concerns or sensitive issues over the telephone. High-risk mothers preferred to have a face-to-face consultation to reduce anxiety. They perceived difficulty in seeking information for help during COVID 19.

5	Bradfield *et al.* [13] Australia 2021	60% (confounders not controlled, response rate not mentioned)	3701	Quantitative	Significantly fewer women were satisfied with the modified maternal health care services during COVID-19. Many claimed they could receive timely and precise answers to their questions about the impact of COVID-19 on maternal care.
6	Temesgen *et al.* [10] Ethiopia 2021	100%	844	Quantitative	Fear of getting COVID-19 while receiving service was frequently mentioned. Transmission from healthcare providers and lack of sanitiser or clean water in the health facility may influence infection.
7	Saso *et al.* [16] 290 members in 51 countries,	60% (no meta-inference made, not	48	Mixed-Method	Mothers perceived uncertainty on the services provided—the majority of services

	including 32 (63%) LMICs and 19 (37%) HICs 2020	adhere to the quality of qualitative component)			suspended from routine antenatal check-ups and vaccination services in some centres.
8	Syed Anwar Aly *et al.* [14] Malaysia 2021	100%	415	Quantitative	This study reported no negative impact of COVID-19 on maternal healthcare services. The majority of the mothers claimed their antenatal appointments were not affected during a pandemic. They perceived that the government clinics or private general practitioners were very supportive and helpful. However, some mothers were anxious when they were not allowed to be accompanied by their partners during antenatal follow-up.

Table 3. Determinants of COVID-19 pandemic on antenatal care service

	Articles

Determinant (Theme)	1	2	3	4	5	6	7	8	Total
	Article Reference ID								
	Altman *et al.* [11]	Ceulemans *et al.* [15]	Hailemariam *et al.* [4]	Karavadra *et al.* [12]	Bradfield *et al.* [13]	Temesgen *et al.* [10]	Saso *et al.* [16]	Syed Anwar Aly *et al.* [14]	
1. Psychosocial Support		*	*						2
2. Poor quality			*		*	*			3
3. Poor virtual Consultation				*					1
4. Failure to meet mothers' demand	*	*					*		3

5. Mother's satisfaction								*	1
Total	1	2	2	1	1	1	1	1	10
