# Supplementary material for: The determinants of maternal perception of antenatal care services during the COVID-19 pandemic critical phase: A systematic review
Source: PLoS One. 2024 Feb 23;19(2):e0297563. doi: 10.1371/journal.pone.0297563 (PMC10889657; doi:10.1371/journal.pone.0297563)
Supplement: S3 Table — (PDF) [file pone.0297563.s003.pdf]

Quality Appraisal: The determinants of maternal perception of antenatal care services during the COVID-19 pandemic critical phase: A systematic review

				MMAT METHODOLOGICAL QUALITY DATA																		
						S1		S2		Criteria according to study design type (refer MMAT 2018)												
No.	Name of data extractor	Author/Year	Title (citation reference : study ID)	Category of study design (refer MMAT flow)	Code MMAT	Are there clear research question?	Comment reviewer	Do the collected data allow to address the research questions?	Comment reviewer	1.1/2.1/3.1/4.1/5.1	Comment reviewer	1.2/2.2/3.2/4.2/5.2	Comment reviewer	1.3/2.3/3.3/4.3/5.3	Comment reviewer	1.4/2.4/3.4/4.4/5.4	Comment reviewer	1.5/2.5/3.5/4.5/5.5	Comment reviewer	Overall comment reviewer	RESULT (RECOMMENDED- Y/N)	Grading
1	NIB	Altman et al. : 2021	Where the System Failed: TheCOVID-19 Pandemic's Impact on Pregnancy and Birth Care	Qualitative	1	YES	-	YES	-	YES	-	YES	-	YES	-	YES	-	YES	-	Appropriate study	YES	5/5 = 100%
2	RS	Syed Anwar Aly et al. : 2021	Pregnancy and COVID-19 Pandemic Perception in Malaysia: A Cross-Sectional Study	Quantitative	4	YES	-	YES	-	YES	-	YES	-	YES	-	YES	-	YES	-	Appropriate study	YES	5/5=100%
3	NIB	Ceuleman et al. : 2021	Vaccine Willingness and Impact of the COVID-19 Pandemic on Women's Perinatal Experiences and Practices—A Multinational, Cross-Sectional Study Covering the FirstWave of the Pandemic	Quantitative	4	YES	-	YES	-	YES	-	YES	-	YES	-	YES	-	YES	-	Appropriate study	YES	5/5 = 100%
4	RS	Goyal et al. : 2021	Effect of the COVID-19 pandemic on maternal health due to delay in seeking health care: Experience from a tertiary center	Quantitative	3	NO	-	NO	-	NO	-	NO	-	NO	-	NO	-	NO	-	Not appropriate as this study was not focus on perception of antenatal mothers	NO (use secondary data- admission to the hospital)	0/5 = 0%
5	NIB	Hailemariam et al. : 2021	Exploring COVID-19 Related Factors Influencing Antenatal Care Services Uptake: A Qualitative Study amongWomen in a Rural Community in Southwest Ethiopia	Qualitative	1	YES	-	YES	-	YES	-	YES	-	YES	-	YES	-	YES	-	Appropriate study	YES	5/5 =100%
6	RS	Karavadra et.al. : 2020	Women's perceptions of COVID-19 and their healthcare experiences: a qualitative thematic analysis of a national survey of pregnant women in the United Kingdom	Qualitative	1	YES	-	YES	-	YES	-	YES	-	YES	-	YES	-	YES	-	Appropriate study	YES	5/5=100%
7	RS	Bradfield et al. : 2021	Experiences of receiving and providing maternity care during the COVID-19 in Australia: A five-cohort cross-sectional comparison	Quantitative	4	YES	-	YES	-	YES	-	YES	-	YES	-	NO	Confounders were not accounted	NO	NO changes occured in the exposure	Appropriate study	YES	3/5=60%
8	NIB	Saso et al. : 2020	Impact of COVID-19 on Immunization Services for Maternal and Infant Vaccines: Results of a Survey Conducted by Imprint—The Immunising Pregnant Women and Infants Network	Mixed Method	1 and 4	YES	-	YES	-	YES	-	YES	-	NO	Only descriptive analysis in quantitative study. For Qualitative study, data collected from free text. No Meta Inference were made	YES	-	NO	Due to the number of respondent is small, the descritive analysis has been applied and free text has been used for qualitative part. Thus, the finding was limited.	Appropriate study	YES	3/5=60%
9	NIB	Temesgen et al. : 2021	Maternal Health Care Services Utilization Amidstcovid-19 Pandemic in West Shoa Zone, Central Ethiopia	Quantitative	3	YES	-	YES	-	YES	-	YES	-	YES	-	YES	-	YES	-	Appropriate study	YES	5/5=100%
10	RS	Davis et al. 2022	Can Positive Mindsets be Protective Against Stress and Isolation Experienced during the COVID 19 Pandemic? A mixed Method Approach of Understanding Emotional Health and Wellbeing Needs of Perinatal Women	Mixed Method	1 and 4		-	YES	-	YES	-	YES	-	YES	-	YES	-	YES		Appropriate study	YES	5/5=100 %
11	NIB	Bankar and Ghosh 2022	Assesing Antenatal Care (ANC) Services during COVID 19 first wave: Insight into decision making in rural India	Qualitative	1	YES	-	YES	-	YES	-	YES	-	YES	-	YES	-	YES	-	Appropriate Study	YES	5/5=100%

Quality Appraisal: The determinants of maternal perception of antenatal care services during the COVID-19 pandemic critical phase: A systematic review

12	RS	Mirzakhani et al. 2022	High Risk Pregnant Women's Experiences of receiving The Prenatal Care in COVID 19 Pandemic: A Qualitative Study	Qualitative	1	YES	-	YES	-	YES	-	YES	-	YES	-	YES	-	YES		Appropriate Study	YES	5/5=100%
13	NIB	Aydin et al. 2022	Expectant parents' perceptions of healthcare and support during COVID 19 in the UK: a thematic analysis	Mixed Method	1 and 4	YES	-	YES	-	YES	-	NO	The participant only involved one pregnant lady. The rest 506 participants were among postpartum mothers	YES	-	YES	-	YES	-	Appropriate Study	YES	4/5=80%
14	RS	Kumru et al. 2022	Expectation of pregnant women for antenatal care services and factors affecting anxiety severity during the COVID 19 Pandemic	Quantitative	4	YES	-	YES	-	YES	-	YES	-	YES	-	YES	-	YES	-	Appropriate Study	YES	5/5=100%
15	NIB	Sullivan et al. 2022	Rural Residents' Perinatal Experiences During the Initial Months of the COVID-19 Pandemic: A Qualitative Study in British Columbia	Qualitative	1	Yes	-	Yes	-	YES	-	YES	-	YES	-	YES	-	YES	-	Appropriate Study	YES	5/5=100%
